# The benefit of reduced serum phosphate levels depends on patient characteristics: a nationwide cohort study

**DOI:** 10.1093/ckj/sfae263

**Published:** 2024-10-04

**Authors:** Shunsuke Goto, Takayuki Hamano, Hideki Fujii, Masatomo Taniguchi, Masanori Abe, Kosaku Nitta, Shinichi Nishi

**Affiliations:** Committee of the Renal Data Registry, Japanese Society for Dialysis Therapy, Tokyo, Japan; Division of Nephrology and Kidney Center, Kobe University Graduate School of Medicine, Kobe, Japan; Department of Nephrology, Nagoya City University, Graduate School of Medical Sciences, Nagoya, Japan; Department of Nephrology, Osaka University Graduate School of Medicine, Suita, Japan; Division of Nephrology and Kidney Center, Kobe University Graduate School of Medicine, Kobe, Japan; Committee of the Renal Data Registry, Japanese Society for Dialysis Therapy, Tokyo, Japan; Fukuoka Renal Clinic, Fukuoka, Japan; Committee of the Renal Data Registry, Japanese Society for Dialysis Therapy, Tokyo, Japan; Division of Nephrology, Hypertension and Endocrinology, Department of Internal Medicine, Nihon University School of Medicine, Tokyo, Japan; Committee of the Renal Data Registry, Japanese Society for Dialysis Therapy, Tokyo, Japan; Department of Medicine, Kidney Center, Tokyo Women's Medical University, Tokyo, Japan; Division of Nephrology and Kidney Center, Kobe University Graduate School of Medicine, Kobe, Japan

**Keywords:** CKD-MBD, hemodialysis, mortality, NNE, phosphate

## Abstract

**Background:**

In cohort studies of hyperphosphatemic hemodialysis patients, reduced serum phosphate levels have been linked to a lower mortality risk. To investigate whether this benefit is influenced by patient characteristics, we calculated the number needed to be exposed (NNE), stratified by patient characteristics.

**Methods:**

In this 9-year cohort study using the nationwide Japanese registry, we enrolled 78 256 hemodialysis patients aged 18 years or older. We investigated the relationship between time-averaged (TA) phosphate levels and mortality due to cardiovascular disease (CVD) using Cox proportional models. We estimated the 1-year NNE for CVD death in patients with baseline serum phosphate levels ≥6.0 mg/dL and exposure to TA phosphate levels decreasing to 3.5–<5.0 mg/dL using mixed-effects Poisson models.

**Results:**

The hazard ratio of CVD mortality decreased linearly with lower serum TA phosphate levels in those with prior atherosclerotic CVD (ACVD) or diabetic nephropathy (DN) but plateaued with serum phosphate <5.0 mg/dL in those without. The hazard ratios (95% confidence interval) for phosphate ≥7.0 mg/dL compared with 3.5–<3.9 mg/dL were 1.58 (1.38–1.81) in those with prior ACVD, 1.91 (1.68–2.17) in those without, 1.87 (1.63–2.16) in those with DN and 1.65 (1.46–1.87) in those without. However, the NNE for one more person to benefit (NNEB) for CVD death was lower in patients with a history of ACVD than in those without (61 vs 118). Patients with DN had lower NNEB than those without (69 vs 113). In patients with TA albumin ≥3.8 g/dL, older patients had lower NNEB, while patients with TA albumin <3.45 g/dL showed no benefit in some groups, including the elderly.

**Conclusions:**

The benefit of intensive phosphate management may be pronounced in patients with a history of ACVD or DN. A comprehensive approach that considers both age and nutritional status may be necessary when managing serum phosphate levels.

## Graphical Abstract

**Graphical Abstract:**
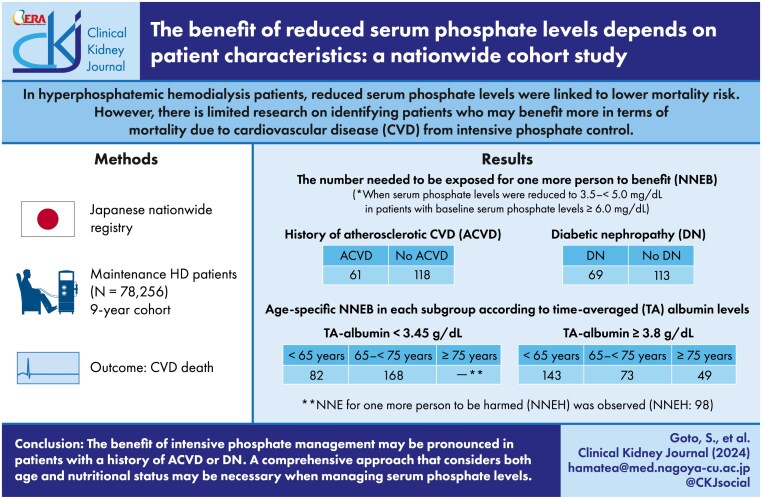

KEY LEARNING POINTS **What was known:**

- In hyperphosphatemic hemodialysis patients, reduced serum phosphate levels were linked to lower mortality risk.
- However, there is limited research seeking to identify patients who may benefit more in terms of mortality due to cardiovascular disease (CVD) from intensive phosphate control.

**This study adds:**

- A positive linear relationship between serum phosphate levels and CVD mortality was found in patients with a history of atherosclerotic CVD (ACVD) or diabetic nephropathy (DN); however, there was no correlation with serum time-averaged (TA) phosphate levels <5.0 mg/dL in those without a history of these diseases.
- The number needed to be exposed for one more person to benefit (NNEB) from CVD death caused by reduced serum phosphate levels was lower in hemodialysis patients with prior ACVD than in those without; patients with DN had lower NNEB compared with those without.
- Among patients with serum TA albumin levels ≥3.8 g/dL, those aged ≥75 years (oldest-old patients) had the lowest NNEB among the age categories.
- On the other hand, patients with serum TA albumin levels <3.45 g/dL showed positive NNE to be harmed (NNEH) was observed in specific groups, including the elderly.

**Potential impact:**

- Intensive management of hyperphosphatemia may benefit hemodialysis patients with a history of ACVD or DN.
- It may be important to adopt a comprehensive approach that considers both age and nutritional status to effectively manage serum phosphate levels.

## INTRODUCTION

Cohort studies on hyperphosphatemic hemodialysis patients have found that reduced serum phosphate levels are associated with a lower risk of mortality [1, 2]. The Evaluate the New Phosphate Iron-Based Binder Sucroferric Oxyhydroxide in Dialysis Patients with the Goal of Advancing the Practice of EBM (EPISODE) trial found that intensive phosphate control can slow the progression of vascular calcification [3]. However, because the EPISODE trial excluded patients without vascular calcification, the benefits of phosphate control may vary according to the degree of vascular calcification. Identifying specific patient subgroups that benefit most from intensive phosphate control is critical, especially given limited healthcare resources. However, little research has been conducted to identify patients who may benefit the most from intensive phosphate control in terms of cardiovascular disease (CVD) mortality.

The number needed to treat (NNT) is a commonly used measure of intervention effects in clinical trials, providing an absolute measure as opposed to relative measures like risk or hazard ratios [4]. For example, if a therapy reduces relative risk by 40% across all subgroups, the NNT for the therapy is 250 in a group with a 1% baseline risk and only 25 in a group with a 10% baseline risk [5]. The Consolidated Standards of Reporting Trials (CONSORT) statement recommends reporting both relative and absolute measures of effect when reporting randomized clinical trials [6]. Although the concept of NNT is more commonly used in clinical trials, it has recently been used in observational studies investigating risk factor associations [7, 8]. The equivalent measure in observational studies is usually referred to as the number needed to be exposed (NNE) because a common situation is the comparison of exposed versus unexposed individuals regarding an adverse event in these studies [9]. Furthermore, the abbreviation, NNE for one more person to be harmed (NNEH) or benefit (NNEB) was used to differentiate between harmful and beneficial exposures. However, no previous study has estimated the NNE for reduction of serum phosphate levels in hemodialysis patients.

This study sought to determine whether the relationship between high serum phosphate levels and CVD or all-cause mortality varies according to patient characteristics. Furthermore, the study determines whether lowering serum phosphate levels benefits or harms hyperphosphatemic dialysis patients. This evaluation will be conducted in various subgroups based on age, sex, history of atherosclerotic CVD (ACVD), renal etiology of diabetic nephropathy (DN), serum alkaline phosphatase (ALP) levels and serum albumin levels.

## MATERIALS AND METHODS

### Data source, study population and study design

We used data from our previous study [10]. This study is a 9-year cohort study using data from the Japanese Society for Dialysis Therapy Renal Data Registry (JRDR). The JRDR is a nationwide registry for dialysis patients in Japan, administered by the Japanese Society for Dialysis Therapy (JSDT). The JSDT collects data through questionnaires distributed to all dialysis facilities in Japan, resulting in a response rate of over 95%. A more detailed description of the survey protocol is available elsewhere [11].

The study population included people who were undergoing maintenance hemodialysis or hemodiafiltration three times per week at the end of 2009. Inclusion criteria involved patients aged 18 years or older, with a treatment duration of 3–6 h per session and a dialysis vintage of 1 year or more. We excluded patients receiving combined therapy with peritoneal dialysis, those who lacked data on calcium, phosphate, intact or whole parathyroid hormone (PTH), and albumin, and those with implausible outcome data (Supplementary data, Fig. S1). The study protocol received approval from the Ethics Committee of the JSDT (approval number 54), which followed the Declaration of Helsinki. The study did not obtain informed consent from the participants, as approved by the Ethics Committee of the JSDT.

### Definition

Serum calcium levels were corrected using the formula: corrected calcium = serum calcium (mg/dL) + [4 – serum albumin (g/dL)], if serum albumin levels were <4 g/dL [12]. Whole PTH levels were multiplied by 1.7 to obtain equivalent values for the intact PTH assay if whole PTH assays were employed [13]. We used data of blood collected before dialysis session at the beginning of week. Geriatric nutritional risk index (GNRI) was calculated by the following equation: GNRI = [14.89 × serum albumin (g/dL)] + [41.7 × body weight (kg)/ideal body weight (kg)]. Ideal body weight was calculated by the following formula: ideal body weight = height (m) × height (m) × 22. If body weight exceeded ideal body weight, body weight/ideal body weight was set to 1 [14, 15]. The performance status was classified using the Eastern Cooperative Oncology Group Performance Status classification [16]. The definition of CVD death has been given elsewhere [10]. In summary, CVD death was defined as death caused by heart failure, pulmonary edema, coronary artery disease (CAD), arrhythmia, endocarditis, valvular disease, other cardiac diseases, subarachnoid hemorrhage, cerebral hemorrhage, cerebral infarction, other cerebrovascular diseases, sudden death, aortic aneurysm or other vascular diseases. The five leading causes of CVD mortality were heart failure, CAD, sudden death, hemorrhagic stroke and cerebral infarction. Deaths from pulmonary edema and arrhythmia were classified as “heart failure” and “sudden death,” respectively. ACVD history was defined as a previous myocardial infarction, cerebral infarction or amputation.

### Statistical analysis

Continuous variables with normal distributions are shown as mean and standard deviation, skewed distributions as median and interquartile range, and categorical variables as number and percentage.

We used multivariate Cox proportional hazard models to investigate the relationship between time-averaged (TA) phosphate levels and CVD or all-cause mortality. The TA model divided the follow-up period into 1-year intervals, with the mean values of variables up to the index time acting as time-varying covariates. Assuming nonlinear relationships, the TA phosphate levels were divided into deciles or modeled using restricted cubic splines (RCS). The number of knots in each model was determined based on the Akaike Information Criterion, and values <2 mg/dL or ≥9 mg/dL of TA phosphate levels were excluded to eliminate outliers in the RCS analysis. Analyses were stratified by age (<65, 65–<75 or ≥75 years), sex, history of ACVD, renal etiology of DN, serum ALP levels (male <225 or ≥225, female <252 or ≥252), or serum TA albumin levels (quintiles: <3.45, 3.45–<3.65, 3.65–<3.8, 3.8–<4.0 or ≥4.0 g/dL). Additive analyses stratifying age and serum TA albumin levels were performed because potential nutritional status may influence different associations across age groups between serum phosphate levels and CVD or all-cause mortality. Furthermore, we investigated the proportions of all-cause death and CVD deaths, as well as the relationship between serum TA phosphate levels and the five leading causes of CVD death, to better understand the mechanism of variation across groups stratified by age and serum TA albumin levels. The proportions of CVD death, death due to CAD, and death due to hemorrhagic stroke were evaluated using a nonparametric test for trends across ordered groups. Moreover, as a sensitivity analyses, we performed analyses replacing serum albumin levels with TA GNRI levels (tertiles: <91.5, 91.5–<96.8 or ≥96.8) as a nutritional marker. The analyses investigated the association between TA phosphate levels and CVD death.

For patients with baseline serum phosphate levels ≥6.0 mg/dL, the 1-year NNEB or NNEH for CVD death or all-cause death was calculated. Exposures were defined as moving from high phosphate levels (≥6.0 mg/dL) to lower phosphate ranges (3.5–<5.0, 5.0–<5.5, 5.5–<6.0 mg/dL). These ranges of serum phosphate levels were determined using the recommended ranges of the Japanese guidelines (3.5–6.0 mg/dL) [13] and findings on the association of TA phosphate levels with CVD death in a previous study [10]. The 1-year NNEB and NNEH were calculated as follows: first, we estimated the adjusted incident rate ratios (IRR) of outcomes across TA phosphate categories [<3.5, 3.5–<5.0, 5.0–<5.5, 5.5–<6.0, ≥6.0 mg/dL (reference)] using mixed-effects Poisson models. Then, we estimated the difference between predicted probabilities of events per person under the factual (maintaining ≥6.0 mg/dL of TA phosphate levels) and counterfactual (decreasing to 3.5–5.0, 5.0–<5.5 or 5.5–<6.0 mg/dL of TA phosphate levels) scenarios [17]. Finally, 1-year NNEs were calculated by taking the reciprocal of the difference.

Adjusted variables in these analyses were age, sex, dialysis vintage, dialysis time, dialysis modality (hemodialysis or hemodiafiltration), body mass index (BMI), Kt/V, normalized protein catabolic rate, primary cause of renal failure (glomerulonephritis, DN, hypertension, polycystic kidney disease, others and unknown), history of myocardial infarction, cerebral hemorrhage, cerebral infarction, amputation, hip fracture, parathyroidectomy (PTx), and percutaneous ethanol injection therapy (PEIT), calcium, intact PTH, albumin, creatinine, C-reactive protein (CRP), hemoglobin, magnesium, ALP, chronic kidney disease–mineral bone disorder related medications [calcium carbonate, sevelamer chloride, lanthanum carbonate, other phosphate binders, oral vitamin D receptor activator (VDRA), intravenous VDRA and cinacalcet], dialysate calcium concentration (<2.75, 2.75–<3.0, ≥3.0 mEq/L and acetate-free) and performance status. Time-varying covariates included age, dialysis time, BMI, Kt/V, normalized protein catabolic rate (nPCR), history of myocardial infarction, cerebral hemorrhage, cerebral infarction, amputation, calcium, intact PTH, albumin, creatinine, CRP and hemoglobin. These time-varying covariates were treated as missing values when the number of hemodialysis sessions per week exceeded three. CRP was log-transformed to normalize the distribution. Serum calcium and intact PTH levels were divided into deciles, while serum magnesium levels were divided into quintiles, based on U-shaped associations between these variables and mortality [18, 19]. Cases with complete baseline data were analyzed because most characteristics between complete and noncomplete cases were similar [10].

A *P*-value <.05 was considered statistically significant. All statistical analyses were conducted using Stata/MP 14.2 software for Windows (Stata, College Station, TX, USA).

## RESULTS

Patients with ACVD were older compared with those without, were predominantly male, and had shorter dialysis vintage and time, lower Kt/V and nPCR, a higher likelihood of having DN and a history of fracture, lower serum phosphate, albumin, creatinine and magnesium levels, higher CRP levels, and a poorer performance status (Table 1). Patients with DN compared with those without were more likely to be male, and had shorter dialysis vintage and time, lower hemodiafiltration usage, lower Kt/V and nPCR, higher prevalence of myocardial infarction, cerebral infarction and amputation, lower likelihood of history of PTx and PEIT, higher BMI, lower serum calcium, PTH and creatinine levels, and a poorer performance status.

**Table 1:** Baseline features of patients with or without a history of ACVD at baseline and patients with or without the renal etiology of DN.

	History of ACVD at baseline		DN	
	ACVD (*N* = 17 442)	No ACVD (*N* = 60 814)	DN (*N* = 26 739)	No DN (*N* = 51 517)
Age (years)	69.5 ± 10.3	64.3 ± 12.7	66.2 ± 10.8	65.1 ± 13.1
Men (%)	67.8	59.6	67.2	58.4
Dialysis vintage (months)	70 (38–122)	79 (41–143)	55 (32–90)	95 (47–170)
Dialysis time (h/week)	11.8 ± 1.4	12.0 ± 1.4	11.8 ± 1.4	12.0 ± 1.4
Hemodiafiltration (%)	6.7	7.5	5.2	8.4
BMI (kg/m^2^)	21.2 ± 4.2	21.3 ± 4.1	22.1 ± 4.3	20.8 ± 3.9
Kt/V	1.40 ± 0.28	1.45 ± 0.29	1.36 ± 0.27	1.48 ± 0.29
nPCR (g/kg/day)	0.85 ± 0.17	0.89 ± 0.17	0.85 ± 0.17	0.90 ± 0.17
Cause of kidney failure (%)				
Glomerulonephritis	28.3	43.3	0.0	60.7
Diabetes	49.4	29.8	100.0	0.0
Hypertension	8.1	6.6	0.0	10.5
Polycystic kidney disease	2.5	3.9	0.0	5.5
Others	6.0	7.6	0.0	11.0
Unknown	5.7	8.8	0.0	12.3
Past history (%)				
Myocardial infarction	34.9	0.0	11.7	5.8
Cerebral hemorrhage	7.5	3.8	4.2	4.9
Cerebral infarction	65.8	0.0	20.0	11.9
Amputation	12.5	0.0	6.2	1.0
Fracture	4.3	2.4	3.2	2.6
PTx	4.4	7.1	1.3	9.2
PEIT	1.0	1.5	0.5	1.8
Laboratory tests				
Corrected calcium (mg/dL)	9.4 ± 0.9	9.4 ± 0.9	9.2 ± 0.8	9.4 ± 0.9
Phosphate (mg/dL)	5.0 ± 1.4	5.2 ± 1.4	5.1 ± 1.4	5.2 ± 1.4
Intact PTH (pg/mL)	118 (61–197)	126 (66–210)	113 (59–187)	130 (68–218)
Albumin (g/dL)	3.6 ± 0.4	3.7 ± 0.4	3.7 ± 0.4	3.7 ± 0.4
TA albumin (g/dL)	3.6 ± 0.4	3.7 ± 0.3	3.7 ± 0.3	3.7 ± 0.3
Creatinine (mg/dL)	9.7 ± 2.7	10.9 ± 2.8	9.9 ± 2.6	11.0 ± 2.9
CRP (mg/dL)	0.1 (0.0–0.5)	0.1 (0.0–0.3)	0.1 (0.0–0.4)	0.1 (0.0–0.3)
Hemoglobin (g/dL)	10.6 ± 1.3	10.7 ± 1.2	10.6 ± 1.2	10.7 ± 1.2
Magnesium (mg/dL)	2.6 ± 0.5	2.6 ± 0.5	2.6 ± 0.5	2.6 ± 0.5
ALP (IU/L)	271 ± 135	261 ± 141	263 ± 134	263 ± 143
Medication (%)				
Calcium carbonate	58.0	64.4	63.3	62.8
Sevelamer	25.9	33.7	26.4	34.9
Lanthanum carbonate	10.9	15.5	12.6	15.4
Other phosphate binders	2.6	4.1	3.0	4.1
Oral VDRA	37.1	39.0	39.2	38.3
Intravenous VDRA	27.0	30.8	23.2	33.5
Cinacalcet	10.9	14.7	7.0	17.4
Dialysate calcium (%)				
<2.75 mEq/L	37.8	38.0	38.7	37.6
2.75–<3.0 mEq/L	8.0	8.6	7.7	8.8
≥3.0 mEq/L	49.9	49.5	49.6	49.5
Acetate-free dialysis	4.3	4.0	4.0	4.1
Performance status (%)				
Grade 0	28.7	51.6	38.8	50.5
Grade 1	28.8	30.9	30.8	30.2
Grade 2	20.5	10.4	15.9	10.9
Grade 3	12.1	4.5	8.6	5.0
Grade 4	10.0	2.7	5.9	3.5

Data are presented as median (interquartile range), mean ± standard deviation or %.

**Figure 1:**
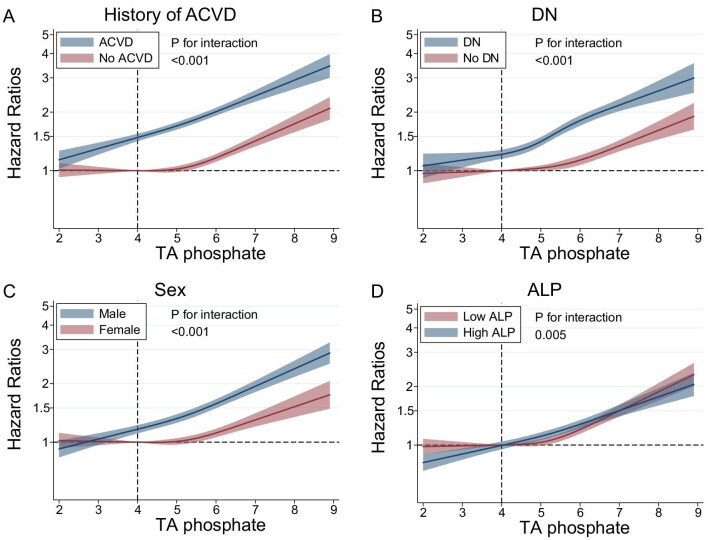
Relationship between TA phosphate levels and cardiovascular mortality in subgroups based on (**A**) history of ACVD, (**B**) DN, (**C**) sex or (**D**) serum ALP levels.

Patients with a history of ACVD, DN, males and high serum ALP levels all showed linear associations (Fig. 1, Table 2). However, the association plateaued with serum TA phosphate levels <5.0 mg/dL in those without a history of ACVD, without DN, females or with low serum ALP levels. Similar trends were seen in the relationship between TA phosphate levels and all-cause mortality (Supplementary data, Fig. S2 and Table S1).

**Table 2:** Hazard ratios and 95% CIs for cardiovascular mortality based on serum TA phosphate levels in subgroups according to the history of ACVD, DN, sex, or serum ALP levels.

	History of ACVD		DN		Sex		ALP	
	ACVD	No ACVD	DN	No DN	Male	Female	Low ALP	High ALP
*P* for the interaction	<.001		<.001		<.001		.040	
<3.5	1.02 (0.91, 1.14)	1.02 (0.91, 1.15)	1.19 (1.05, 1.35)*	0.88 (0.79, 0.98)*	1.01 (0.91, 1.12)	1.01 (0.89, 1.14)	1.09 (0.95, 1.24)	0.98 (0.89, 1.09)
3.5–<3.9	ref	ref	ref	ref	ref	ref	ref	ref
3.9–<4.3	1.11 (1.00, 1.24)	1.02 (0.92, 1.14)	1.12 (0.99, 1.26)	1.03 (0.93, 1.14)	1.09 (0.99, 1.20)	1.03 (0.91, 1.16)	1.09 (0.97, 1.24)	1.05 (0.96, 1.16)
4.3–<4.7	1.06 (0.96, 1.18)	1.01 (0.91, 1.13)	1.12 (1.00, 1.25)	0.99 (0.90, 1.09)	1.09 (0.99, 1.19)	0.98 (0.87, 1.10)	1.07 (0.95, 1.20)	1.02 (0.93, 1.13)
4.7–<5.1	1.10 (0.99, 1.21)	1.07 (0.96, 1.18)	1.12 (1.00, 1.26)*	1.05 (0.96, 1.15)	1.07 (0.98, 1.18)	1.11 (1.00, 1.25)	1.07 (0.95, 1.20)	1.10 (1.01, 1.21)*
5.1–<5.4	1.13 (1.02, 1.26)*	1.22 (1.10, 1.36)*	1.33 (1.18, 1.49)*	1.08 (0.97, 1.19)	1.25 (1.13, 1.37)*	1.11 (0.98, 1.25)	1.15 (1.02, 1.29)*	1.22 (1.11, 1.35)*
5.4–<5.8	1.21 (1.09, 1.34)*	1.23 (1.11, 1.37)*	1.32 (1.18, 1.48)*	1.15 (1.04, 1.27)*	1.31 (1.19, 1.45)*	1.12 (0.99, 1.26)	1.27 (1.13, 1.43)*	1.21 (1.09, 1.33)*
5.8–<6.3	1.26 (1.13, 1.41)*	1.30 (1.16, 1.45)*	1.42 (1.26, 1.60)*	1.18 (1.06, 1.30)*	1.33 (1.20, 1.47)*	1.24 (1.09, 1.41)*	1.35 (1.20, 1.53)*	1.25 (1.13, 1.39)*
6.3–<7.0	1.34 (1.19, 1.52)*	1.50 (1.33, 1.68)*	1.47 (1.30, 1.68)*	1.38 (1.23, 1.54)*	1.48 (1.33, 1.65)*	1.39 (1.21, 1.59)*	1.52 (1.33, 1.73)*	1.38 (1.23, 1.54)*
7.0–	1.58 (1.38, 1.81)*	1.91 (1.68, 2.17)*	1.87 (1.63, 2.16)*	1.65 (1.46, 1.87)*	1.91 (1.70, 2.15)*	1.55 (1.33, 1.81)*	1.96 (1.70, 2.26)*	1.63 (1.44, 1.85)*

*P < .05.

**Figure 2:**
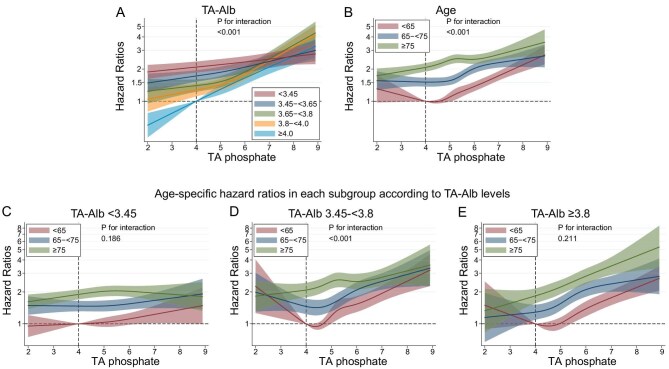
Relationship between TA phosphate and cardiovascular mortality in subgroups based on age or serum TA albumin (TA-Alb) levels. (**A**) TA-Alb, (**B**) age, (**C**) age among patients with serum TA-Alb levels <3.45 g/dL, (**D**) age among patients with serum TA-Alb levels of 3.45–<3.8 g/dL and (**E**) age among patients with serum TA-Alb levels ≥3.8 g/dL.

Stratified analysis by serum TA albumin category revealed a strong association between high serum TA phosphate levels and CVD mortality in patients with the highest category of serum TA albumin levels (≥4.0 g/dL), which weakened as serum TA albumin levels decreased (Fig. 2A, Table 3). A similar trend was observed in stratified analysis by TA GNRI category (Supplementary data, Fig. S3A). Although older patients showed a weaker association compared with younger patients (Fig. 2B, Table 3), the associations of increased serum TA phosphate levels (≥4.0 mg/dL) with CVD mortality across age categories were comparable when stratified by serum TA albumin category (Fig. 2C–E, Table 4). Hypophosphatemia was linked to CVD mortality in patients aged <65 years with serum TA albumin levels >3.45 g/dL (Fig. 2D–E). This was not seen with all-cause mortality (Supplementary data, Fig. S4, and Tables S2 and S3). There was no substantial difference found in any other association between CVD and all-cause mortality.

**Table 3:** Hazard ratios and 95% CIs for cardiovascular mortality based on serum TA phosphate levels in subgroups according to age or serum TA albumin levels.

	TA albumin					Age		
	<3.45	3.45–<3.65	3.65–>3.8	3.8–<4.0	≥4.0	<65	65–<75	≥75
*P* for the interaction	<.001					<.001		
<3.5	0.98 (0.88, 1.09)	1.07 (0.88, 1.29)	1.04 (0.79, 1.38)	1.18 (0.86, 1.62)	0.94 (0.58, 1.52)	0.90 (0.71, 1.15)	1.11 (0.95, 1.30)	0.99 (0.89, 1.10)
3.5–<3.9	ref	ref	ref	ref	ref	ref	ref	ref
3.9–<4.3	1.07 (0.96, 1.19)	1.02 (0.87, 1.20)	0.91 (0.73, 1.14)	1.29 (1.01, 1.66)	1.14 (0.79, 1.63)	0.91 (0.73, 1.13)	1.06 (0.91, 1.22)	1.09 (0.99, 1.20)
4.3–<4.7	1.02 (0.92, 1.14)	1.08 (0.93, 1.26)	0.91 (0.73, 1.12)	1.16 (0.91, 1.48)	1.18 (0.83, 1.68)	0.80 (0.65, 0.99)	1.03 (0.90, 1.18)	1.08 (0.98, 1.18)
4.7–<5.1	1.05 (0.95, 1.17)	1.13 (0.97, 1.31)	1.03 (0.84, 1.26)	1.22 (0.96, 1.54)	1.17 (0.83, 1.65)	0.94 (0.77, 1.14)	1.01 (0.88, 1.16)	1.14 (1.04, 1.25)*
5.1–<5.4	1.03 (0.91, 1.15)	1.24 (1.06, 1.45)*	1.21 (0.98, 1.49)	1.43 (1.13, 1.82)*	1.58 (1.12, 2.23)*	0.99 (0.81, 1.22)	1.18 (1.03, 1.36)*	1.21 (1.10, 1.34)*
5.4–<5.8	1.12 (1.00, 1.26)	1.28 (1.10, 1.50)*	1.20 (0.97, 1.48)	1.44 (1.14, 1.83)*	1.55 (1.10, 2.19)*	1.21 (1.00, 1.48)	1.23 (1.07, 1.42)*	1.17 (1.05, 1.29)*
5.8–<6.3	1.08 (0.95, 1.23)	1.20 (1.01, 1.41)*	1.39 (1.12, 1.72)*	1.60 (1.26, 2.03)*	1.96 (1.39, 2.76)*	1.13 (0.93, 1.38)	1.43 (1.24, 1.65)*	1.18 (1.05, 1.32)*
6.3–<7.0	1.11 (0.96, 1.27)	1.52 (1.28, 1.81)*	1.60 (1.27, 2.01)*	1.75 (1.37, 2.24)*	1.95 (1.37, 2.78)*	1.45 (1.18, 1.78)*	1.48 (1.27, 1.73)*	1.25 (1.10, 1.43)*
7.0–	1.17 (0.99, 1.39)	1.61 (1.32, 1.98)*	2.12 (1.66, 2.71)*	2.54 (1.96, 3.28)*	2.57 (1.80, 3.69)*	1.94 (1.58, 2.38)*	1.60 (1.35, 1.91)*	1.41 (1.20, 1.65)*

*P < .05.

**Table 4:** Age-specific hazard ratios and 95% CIs for cardiovascular mortality according to serum TA phosphate levels in the subgroups according to serum TA albumin levels.

	TA albumin <3.45			TA albumin 3.45–<3.8			TA albumin ≥3.8		
	<65	65–<75	≥75	<65	65–<75	≥75	<65	65–<75	≥75
*P* for the interaction	.247			<.001			.176		
<3.5	0.86 (0.60, 1.24)	1.01 (0.82, 1.25)	0.97 (0.85, 1.10)	0.95 (0.61, 1.50)	1.28 (0.95, 1.71)	1.01 (0.82, 1.25)	1.37 (0.81, 2.33)	1.21 (0.77, 1.91)	1.01 (0.68, 1.52)
3.5–<3.9	ref	ref	ref	ref	ref	ref	ref	ref	ref
3.9–<4.3	0.87 (0.59, 1.28)	1.10 (0.88, 1.36)	1.07 (0.94, 1.21)	0.72 (0.49, 1.06)	1.02 (0.80, 1.30)	1.03 (0.87, 1.23)	1.22 (0.80, 1.85)	1.14 (0.79, 1.64)	1.34 (0.99, 1.83)
4.3–<4.7	0.96 (0.67, 1.37)	0.97 (0.79, 1.20)	1.03 (0.90, 1.16)	0.67 (0.47, 0.95)*	1.07 (0.85, 1.34)	1.09 (0.93, 1.28)	0.90 (0.59, 1.35)	1.20 (0.85, 1.70)	1.32 (0.98, 1.79)
4.7–<5.1	0.99 (0.70, 1.41)	1.00 (0.81, 1.23)	1.06 (0.93, 1.20)	0.89 (0.65, 1.23)	1.00 (0.80, 1.25)	1.21 (1.03, 1.42)*	1.05 (0.71, 1.55)	1.27 (0.90, 1.77)	1.27 (0.94, 1.72)
5.1–<5.4	0.97 (0.67, 1.43)	0.90 (0.71, 1.14)	1.07 (0.92, 1.23)	0.91 (0.65, 1.27)	1.32 (1.05, 1.66)*	1.31 (1.11, 1.56)*	1.20 (0.81, 1.78)	1.60 (1.14, 2.25)*	1.59 (1.17, 2.16)*
5.4–<5.8	1.30 (0.91, 1.85)	1.10 (0.87, 1.38)	1.07 (0.92, 1.24)	1.27 (0.93, 1.74)	1.33 (1.06, 1.67)*	1.19 (1.00, 1.41)*	1.26 (0.86, 1.86)	1.54 (1.10, 2.15)*	1.58 (1.17, 2.14)*
5.8–<6.3	1.01 (0.69, 1.47)	1.13 (0.89, 1.43)	1.02 (0.87, 1.21)	1.05 (0.76, 1.46)	1.50 (1.19, 1.89)*	1.22 (1.02, 1.47)*	1.41 (0.96, 2.08)	2.01 (1.44, 2.81)*	1.71 (1.25, 2.35)*
6.3–<7.0	1.27 (0.85, 1.88)	1.22 (0.95, 1.58)	0.97 (0.79, 1.19)	1.41 (1.02, 1.95)*	1.83 (1.43, 2.33)*	1.36 (1.11, 1.68)*	1.73 (1.17, 2.55)*	1.67 (1.17, 2.39)*	1.91 (1.37, 2.67)*
7.0–	1.81 (1.22, 2.68)*	1.01 (0.74, 1.39)	1.02 (0.79, 1.32)	1.72 (1.23, 2.39)*	2.03 (1.54, 2.68)*	1.35 (1.03, 1.77)*	2.36 (1.60, 3.50)*	2.10 (1.44, 3.07)*	2.74 (1.90, 3.95)*

*P < .05.

Higher serum TA albumin categories were associated with a higher proportion of CVD deaths, as well as CAD and hemorrhagic stroke deaths (Supplementary data, Fig. S5). Regarding risk of five causes of CVD death, linear associations with serum TA phosphate levels were observed except for hemorrhagic stroke.

**Figure 3:**
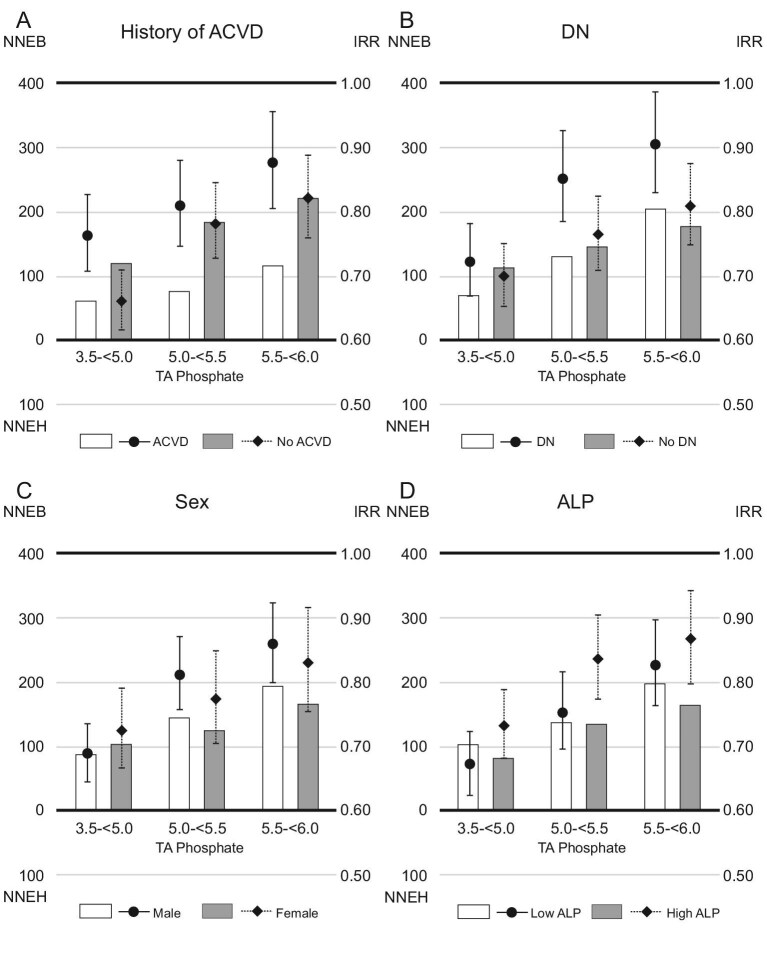
The 1-year NNE and IRR for cardiovascular death in subgroups based on (**A**) history of ACVD, (**B**) DN, (**C**) sex or (**D**) ALP levels. Patients whose serum TA phosphate levels maintained ≥6.0 mg/dL during the study were referenced in the analysis of IRR. The NNEB or NNEH in patients with baseline serum phosphate levels ≥6.0 mg/dL was calculated considering the exposure of TA phosphate levels decreasing to each range (3.5–<5.0, 5.0–<5.5 or 5.5–<6.0 mg/dL).

**Figure 4:**
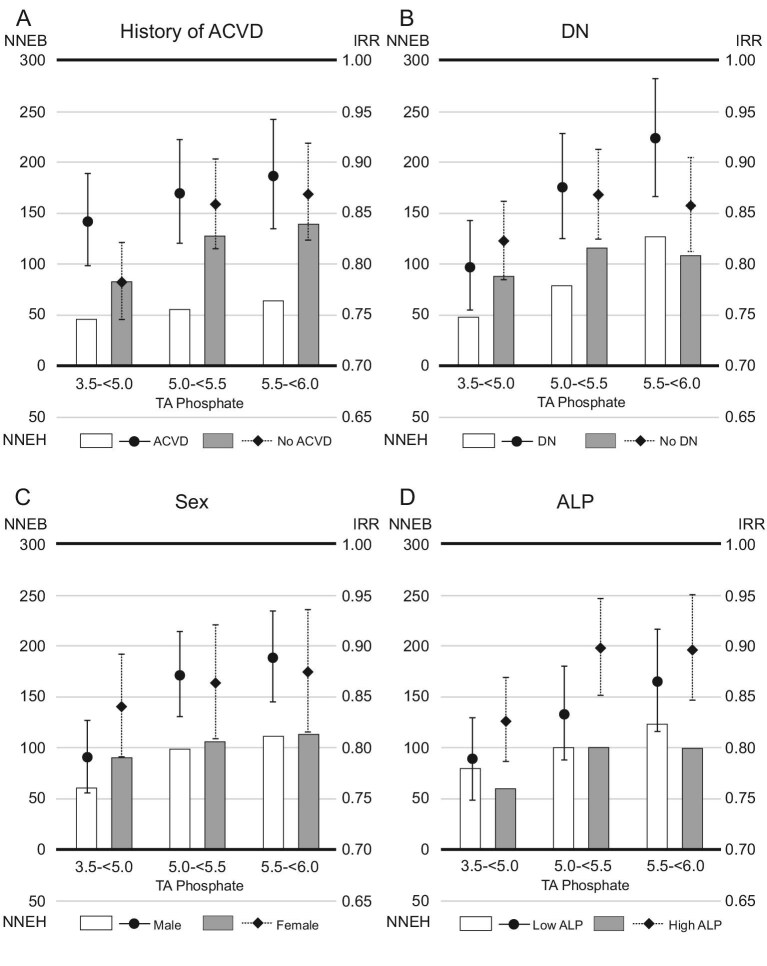
The 1-year NNE and IRR for all-cause death in subgroups based on (**A**) history of ACVD, (**B**) DN, (**C**) sex or (**D**) serum ALP levels. Patients whose serum TA phosphate levels remained ≥6.0 mg/dL during the study were referenced in the analysis of IRR. The NNEB or NNEH in patients with baseline serum phosphate levels ≥6.0 mg/dL was calculated considering exposure of TA phosphate levels decreasing to each range (3.5–<5.0, 5.0–<5.5 or 5.5–<6.0 mg/dL).

The IRRs were higher in patients with a history of ACVD than in those without [IRR (95% confidence interval, 95% CI); 0.77 (0.71, 0.83) vs 0.66 (0.62, 0.71), when serum phosphate levels were reduced to 3.5–<5.0 mg/dL]. However, the 1-year NNEB was lower in patients with a history of ACVD than in those without (NNEB; 61 vs 118, when serum phosphate levels were reduced to 3.5–<5.0 mg/dL) (Fig 3). When serum phosphate levels were reduced to 3.5–<5.0 mg/dL, patients with DN had lower 1-year NNEB than those without DN (NNEB; 69 vs 113), despite having higher IRRs [IRR (95% CI); 0.72 (0.67, 0.78) vs 0.70 (0.65, 0.75)]. The 1-year NNEBs were comparable in subgroups based on sex (NNEB; male vs female, 87 vs 103, when serum phosphate levels were reduced to 3.5–<5.0 mg/dL) or ALP (NNEB; low ALP vs high ALP, 103 vs 82, when serum phosphate levels were reduced to 3.5–<5.0 mg/dL). Similar trends emerged in the analysis of 1-year NNEs and IRRs for all-cause mortality (Fig. 4).

**Figure 5:**
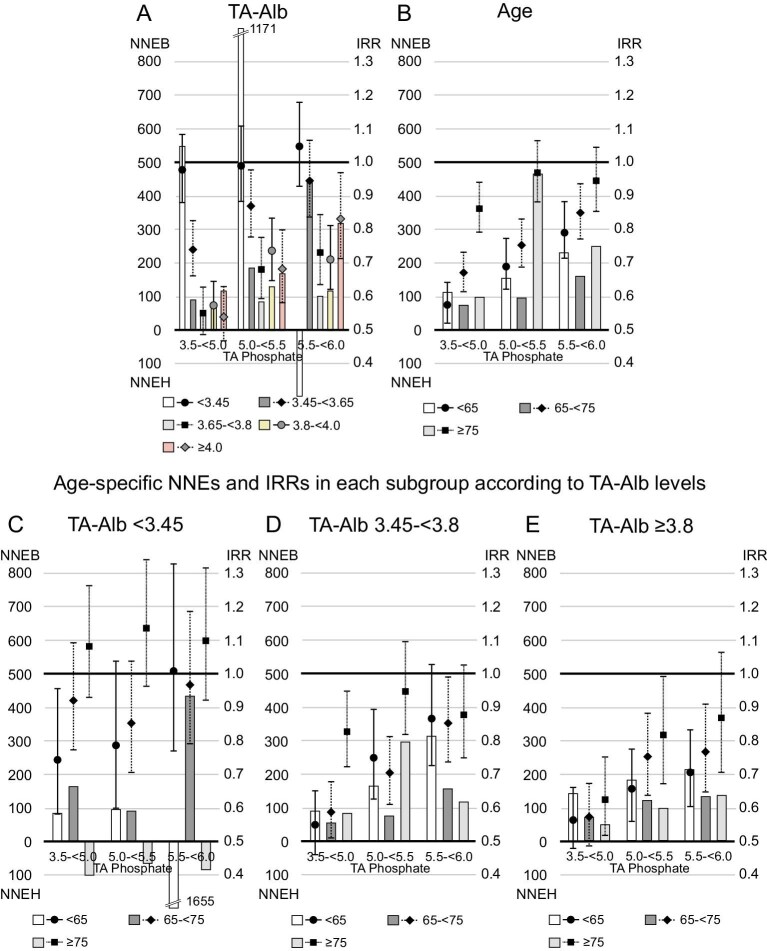
The 1-year NNE and IRR for cardiovascular death in subgroups according to serum TA albumin (TA-Alb) levels or age. Patients whose serum TA phosphate levels maintained ≥6.0 mg/dL during the study were referenced in the analysis of IRR. The NNEB or NNEH in patients with baseline serum phosphate levels ≥6.0 mg/dL was computed under the exposure of TA phosphate levels decreasing to each range (3.5–<5.0, 5.0–<5.5 or 5.5–<6.0 mg/dL). (**A**) TA-Alb, (**B**) age, (**C**) age among patients with serum TA-Alb levels <3.45 g/dL, (**D**) age among patients with serum TA-Alb levels of 3.45–<3.8 g/dL and (**E**) age among patients with serum TA-Alb levels ≥3.8 g/dL.

**Figure 6:**
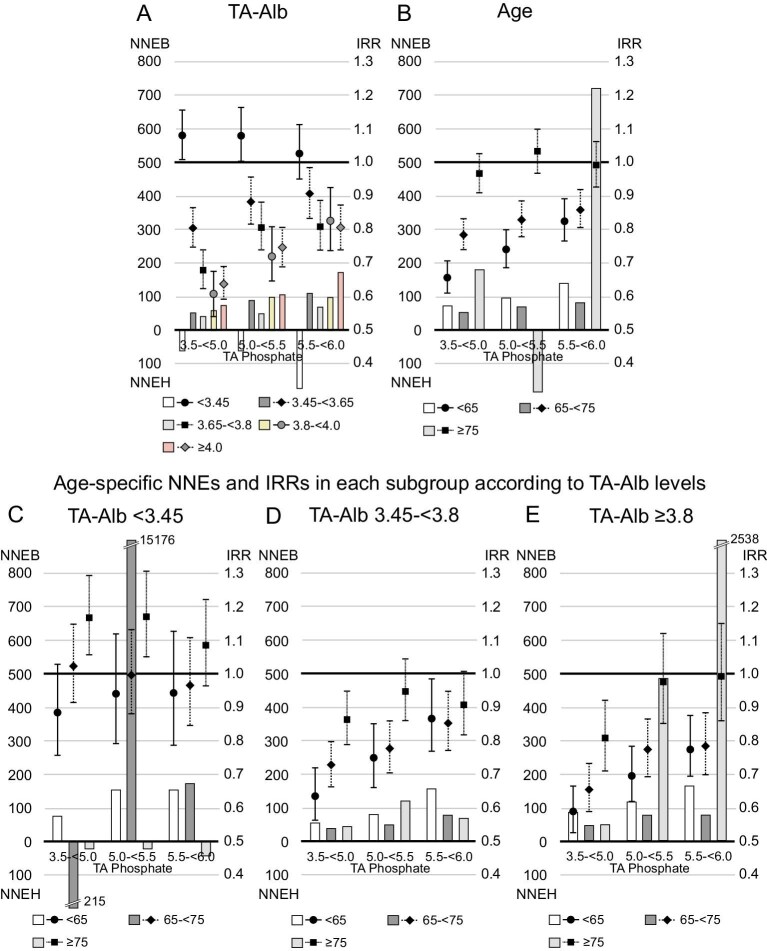
The 1-year NNE and IRR for all-cause mortality in subgroups based on serum TA albumin (TA-Alb) levels or age. Patients whose serum TA phosphate levels maintained ≥6.0 mg/dL during the study were referenced in the analysis of IRR. The NNEB or NNEH in individuals with baseline serum phosphate levels ≥6.0 mg/dL was calculated under the exposure of TA phosphate levels decreasing to each range (3.5–<5.0, 5.0–<5.5 or 5.5–<6.0 mg/dL). (**A**) TA-Alb, (**B**) age, (**C**) age among patients with serum TA-Alb levels <3.45 g/dL, (**D**) age among patients with serum TA-Alb levels of 3.45–<3.8 g/dL and (**E**) age among patients with serum TA-Alb levels ≥3.8 g/dL.

Serum albumin levels showed a U-shaped relationship with 1-year NNEB (Fig. 5). One-year NNEB was lowest in patients with serum TA albumin levels between 3.65–<3.8 g/dL [NNEB in each category of serum TA albumin levels (<3.45, 3.45–<3.65, 3.65–<3.8, 3.8–<4.0 or ≥4.0 g/dL); 549, 92, 60, 80 and 117, when serum phosphate levels were reduced to 3.5–<5.0 mg/dL]. Similar trend was observed in stratified analysis by TA GNRI category (NNEB in each TA GNRI category [<91.5, 91.5–<96.8 or ≥96.8]; 107, 82 and 102, when serum phosphate levels were reduced to 3.5–<5.0 mg/dL, Supplementary data, Fig. S3B). Furthermore, we found a U-shaped relationship between age category and 1-year NNEB. Patients aged 65–<75 years (young-old patients) had the lowest 1-year NNEB [NNEB in each age category (<65, 65–<75 or ≥75 years); 113, 73 and 100, when serum phosphate levels were reduced to 3.5–<5.0 mg/dL]. In patients with serum TA albumin levels ≥3.8 g/dL, however, a higher age category was linked to lower 1-year NNEB; patients aged ≥75 years (oldest-old patients) had the lowest NNEB. Patients with serum TA albumin levels <3.45 g/dL showed 1-year NNEH in some groups, including the oldest-old patients. In stratified analysis by TA GNRI category, similar trend was observed (Supplementary data, Fig. S7). Regarding 1-year NNEs for all-cause death, in patients with TA albumin levels <3.45 g/dL, young patients had a 1-year NNEB <200, whereas young-old patients and oldest-old patients had positive 1-year NNEH or extremely high 1-year NNEB (Fig. 6).

## DISCUSSION

In this large cohort study based on a nationwide Japanese database, the investigation revealed that the association between high serum phosphate levels and mortality varied by age, serum TA albumin levels, history of ACVD or DN. Furthermore, NNEB was lower in patients with a history of ACVD or DN, despite their higher IRRs. Notably, in patients with high serum albumin levels, the elderly had lower NNEB for CVD death, whereas in patients with low serum albumin, positive NNEH was observed in some groups, including elderly patients.

We found a stronger relationship between elevated serum TA phosphate levels and CVD mortality in patients with higher serum TA albumin levels. Previous research has found that high serum phosphate levels are more strongly associated with CVD death (particularly CAD) than other causes [20, 21]. Because higher proportions of CAD deaths were observed in patients with higher categories of serum TA albumin, the difference in the strength of the relationship could be attributed to variations in the cause of CVD death.

The association between elevated phosphate levels and all-cause mortality was similar and stronger in patients with higher serum TA albumin levels. The proportion of CVD among causes of death was relatively lower in malnourished patients, regardless of age. The observed weaker association between elevated phosphate levels and all-cause mortality in this population is not surprising, given that deranged phosphate levels can explain some CVD cases.

Our findings revealed that hypophosphatemia (<4 mg/dL) was linked to an increased risk of CVD mortality in young patients with high serum TA albumin levels. There was no association found with all-cause mortality. The elevated CVD mortality risk linked to hypophosphatemia may be attributed to the high proportion of deaths caused by hemorrhagic stroke in these patients (blue columns in the age category <65 years, Supplementary data, Fig. S4B), as hypophosphatemia tended to be associated with an increased risk of death from hemorrhagic stroke (Supplementary data, Fig. S5D). A previous study found that low serum phosphate levels [<0.9 mmolL (2.78 mg/dL)] at admission were associated with higher mortality among patients with spontaneous intracerebral hemorrhage [22]. In that study, the authors hypothesized that hypophosphatemia could impair brain energy metabolism and cause brain injury.

Our findings highlight the importance of taking both relative and absolute measures when determining the clinical benefit or harm of exposures. Relative measures like hazard ratios, relative risk and IRR are frequently used in observational studies. However, using only relative measures may be insufficient when comparing the effects of exposure in groups with different baseline risks [5]. NNE is an absolute measure that includes baseline risk without exposure, allowing comparisons between groups with different baseline risks. The lower NNEBs observed in patients with a history of ACVD or DN can be attributed to their higher baseline risk. This finding suggests that intensive management of hyperphosphatemia may be more beneficial in these patient populations.

Age may influence the relationship between serum phosphate levels and mortality [23], but our findings suggest that nutritional status may also play a role in the variations observed across age groups. While a U-shaped relationship between age category and NNEB for CVD death was found, this relationship changed when stratified by serum TA albumin levels. Among patients with serum TA albumin levels ≥3.8 g/dL or TA GNRI ≥96.8, the oldest-old patients had the lowest NNEB, while malnourished oldest-old patients (with serum TA albumin <3.45 g/dL or TA GNRI <91.5) showed a positive NNEH if serum TA phosphate levels decreased. This is because the decrease in phosphate levels among malnourished patients may indicate further deterioration of their nutritional status. Our findings highlight the importance of using a comprehensive approach that considers both age and nutritional status, rather than just age, when managing serum phosphate levels.

This study recognizes several limitations. First, because this is an observational study, causal conclusions cannot be drawn. The observational nature of our study necessitates additional interventional studies of strict phosphate control to compare the NNT of this intervention across different subgroups. Second, despite our adjustment for significant confounders, there is still the possibility of residual confounding. Third, our findings cannot be extrapolated to other countries because the mortality rate of Japanese hemodialysis patients was significantly lower than that of Western countries in the Dialysis Outcomes and Practice Patterns Study (DOPPS) study [24]. The study's strengths include a large sample size, a long follow-up period, and the use of both relative and absolute measures to assess associations with mortality across different subgroups.

Finally, the study suggests that NNEB under exposure to decreased phosphate levels is lower in hemodialysis patients with a history of ACVD or DN compared with those without. These findings support intensive management of hyperphosphatemia, particularly in patients with a history of ACVD or DN. Furthermore, because the extent of benefit associated with reduced phosphate levels depends on serum albumin levels in the oldest-old patients, a multifaceted approach that considers both age and nutritional status may be necessary when managing serum phosphate levels. Additional interventional studies are required to validate and establish causation in these findings.

## Supplementary Material

sfae263_Supplemental_File

## Data Availability

The data supporting the findings of this study are available from JSDT, but there are restrictions on their availability because they were used under license for the current study and are therefore not publicly available. The data are available from the authors upon reasonable request and with the permission of the JSDT.
