# Correction to: The benefit of reduced serum phosphate levels depends on patient characteristics: a nationwide cohort study

**DOI:** 10.1093/ckj/sfaf176

**Published:** 2025-06-10

This is a correction to: Shunsuke Goto, Takayuki Hamano, Hideki Fujii, Masatomo Taniguchi, Masanori Abe, Kosaku Nitta, Shinichi Nishi, The benefit of reduced serum phosphate levels depends on patient characteristics: a nationwide cohort study, *Clinical Kidney Journal*, Volume 17, Issue 10, October 2024, sfae263, https://doi.org/10.1093/ckj/sfae263

The following changes have been made to the originally published manuscript. The word “prospective” has been removed from the body of the article, pursuant to a notice issued by the Japanese Society for Dialysis Therapy (JSDT) on March 18, 2025 recommending that researchers not use the term “prospective” for studies using the JSDT Renal Data Registry.

