# Supplementary material for: The benefit of reduced serum phosphate levels depends on patient characteristics: a nationwide cohort study
Source: Clin Kidney J. 2024 Oct 4;17(10):sfae263. doi: 10.1093/ckj/sfae263 (PMC11462437; doi:10.1093/ckj/sfae263)
Supplement: sfae263_Supplemental_File [file sfae263_supplemental_file.pdf]

# Supplementary materials

**Contents:**

**Supplementary Figure 1:** Flow diagram. JRDR, the Japanese Society for Dialysis Therapy Renal Data Registry; HD, hemodialysis; HDF, hemodiafiltration; Ca, calcium; P, phosphate; PTH, parathyroid hormone.

**Supplementary Figure 2:** Relationship between time-averaged (TA) phosphate and all-cause mortality in subgroups according to (A) the history of the atherosclerotic cardiovascular disease (ACVD), (B) diabetic nephropathy (DN), (C) sex, or (D) serum alkaline phosphatase (ALP) levels.

**Supplementary Figure 3:** (A) Relationship between time-averaged (TA) phosphate and (A) cardiovascular death according to TA geriatric nutritional risk index (TA-GNRI) levels. (B) The one-year number needed to be exposed (NNE) and incidence rate ratio (IRR) for cardiovascular death in subgroups according to TA-GNRI levels. Patients whose serum TA phosphate levels maintained ≥6.0 mg/dL during the study were referenced in the analysis of IRR. The NNE to benefit (NNEB) or NNE to be harmed (NNEH) in individuals with baseline serum phosphate levels ≥6.0 mg/dL was calculated under the exposure of TA phosphate levels decreasing to each range (3.5–<5.0, 5.0–<5.5, or 5.5–<6.0 mg/dL).

**Supplementary Figure 4:** Relationship between time-averaged (TA) phosphate and all-cause mortality in subgroups according to age or serum time-averaged albumin (TA-Alb) levels. (A) TA-Alb, (B) age, (C) age among patients with serum TA-Alb levels <3.45 g/dL, (D) age among patients with serum TA-Alb levels of 3.45–<3.8 g/dL, and (E) age among patients with serum TA-Alb levels ≥3.8 g/dL.

**Supplementary Figure 5:** Proportion of (A) all-cause death and (B) cardiovascular death in subgroups stratified age and serum time-averaged albumin (TA-Alb) levels. Range of quintiles of serum TA-Alb levels, as follows: Q1, <3.45 g/dL; Q2, 3.45–<3.65; Q3, 3.65–<3.8; Q4, 3.8–<4.0; and Q5, ≥4.0.

**Supplementary Figure 6.** Relationship between serum time-averaged (TA) phosphate and five main causes of cardiovascular death in all patients. (A) heart failure, (B) coronary artery disease, (C) sudden death, (D) hemorrhagic stroke, and (E) cerebral infarction.

**Supplementary Figure 7:** The one-year number needed to be exposed (NNE) and incidence rate ratio (IRR) for cardiovascular death in subgroups according to age and TA geriatric nutritional risk index (GNRI) levels. Patients whose serum time-averaged (TA) phosphate levels maintained ≥6.0 mg/dL during the study were referenced in the analysis of IRR. The NNE to benefit (NNEB) or NNE to be harmed (NNEH) in individuals with baseline serum phosphate levels ≥6.0 mg/dL was calculated under the exposure of TA phosphate levels decreasing to each range (3.5–<5.0, 5.0–<5.5, or 5.5–<6.0 mg/dL). (A) age among patients with TA-GNRI levels <91.5, (B) age among patients with TA-GNRI levels of 91.5–<96.8, and (C) age among patients with TA-GNRI levels ≥96.8.

**Supplementary Table 1:** Hazard ratios and 95% confidence intervals for all-cause mortality

based on serum time-averaged phosphate levels in subgroups according to the history of atherosclerotic cardiovascular disease (ACVD), diabetic nephropathy (DN), sex, and serum alkaline phosphatase (ALP) levels.

**Supplementary Table 2:** Hazard ratios and 95% confidence intervals for all-cause mortality based on serum time-averaged phosphate levels in subgroups according to age and serum time-averaged albumin levels.

**Supplementary Table 3:** Age-specific hazard ratios and 95% confidence intervals for all-cause mortality based on serum time-averaged phosphate levels in subgroups according to serum time-averaged albumin levels.

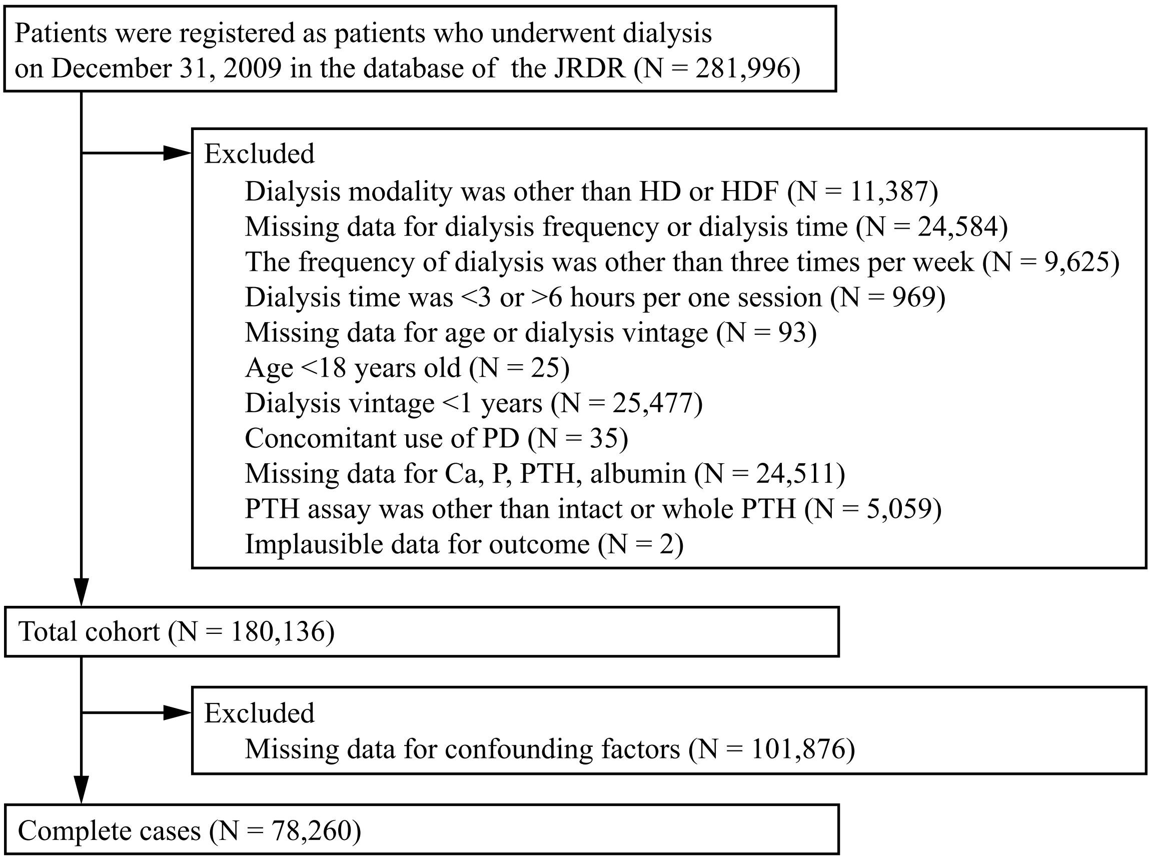


**Supplementary Figure 1.** Flow diagram. JRDR, the Japanese Society for Dialysis Therapy Renal Data Registry; HD, hemodialysis; HDF, hemodiafiltration; Ca, calcium; P, phosphate; PTH, parathyroid hormone.

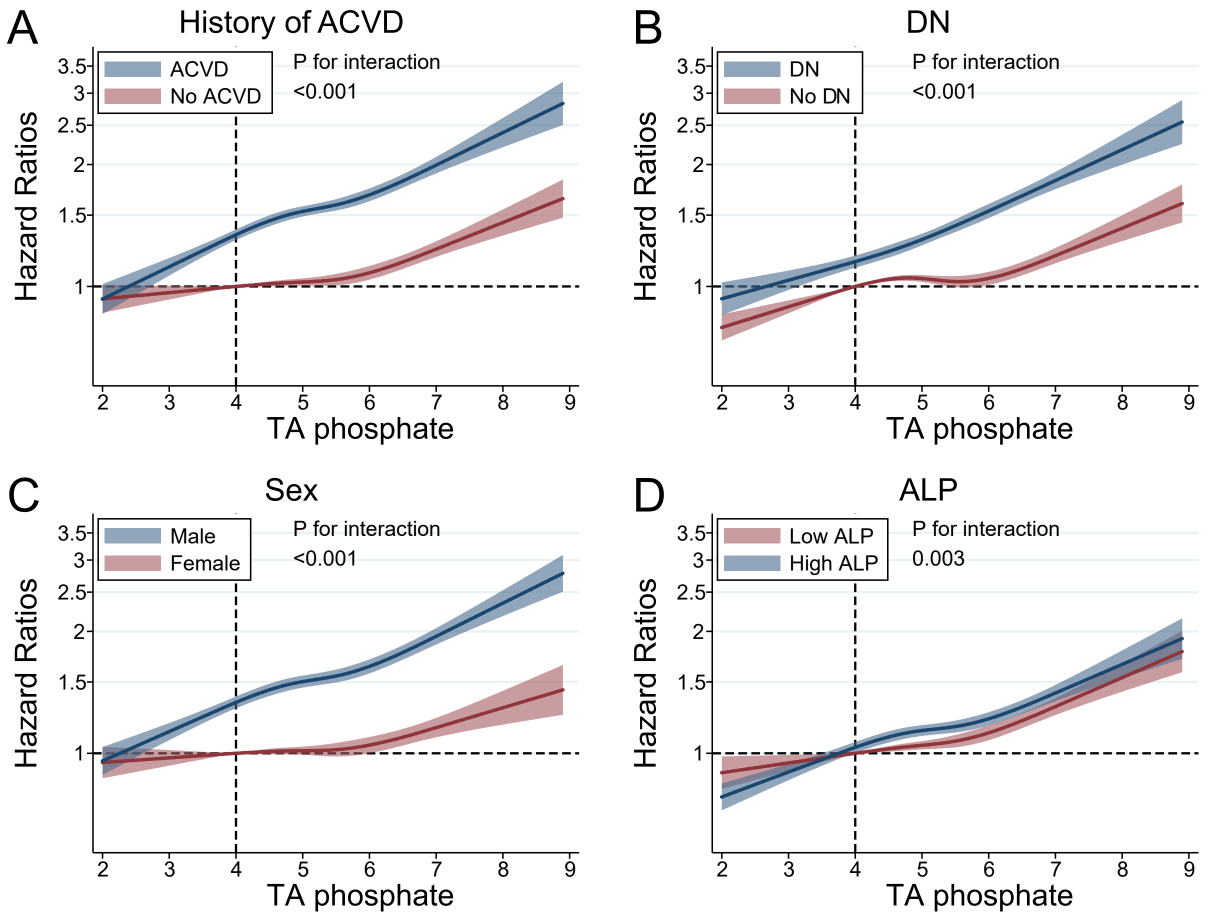


**Supplementary Figure 2.** Relationship between time-averaged (TA) phosphate and all-cause mortality in subgroups according to (A) the history of the atherosclerotic cardiovascular disease (ACVD), (B) diabetic nephropathy (DN), (C) sex, or (D) serum alkaline phosphatase (ALP) levels.

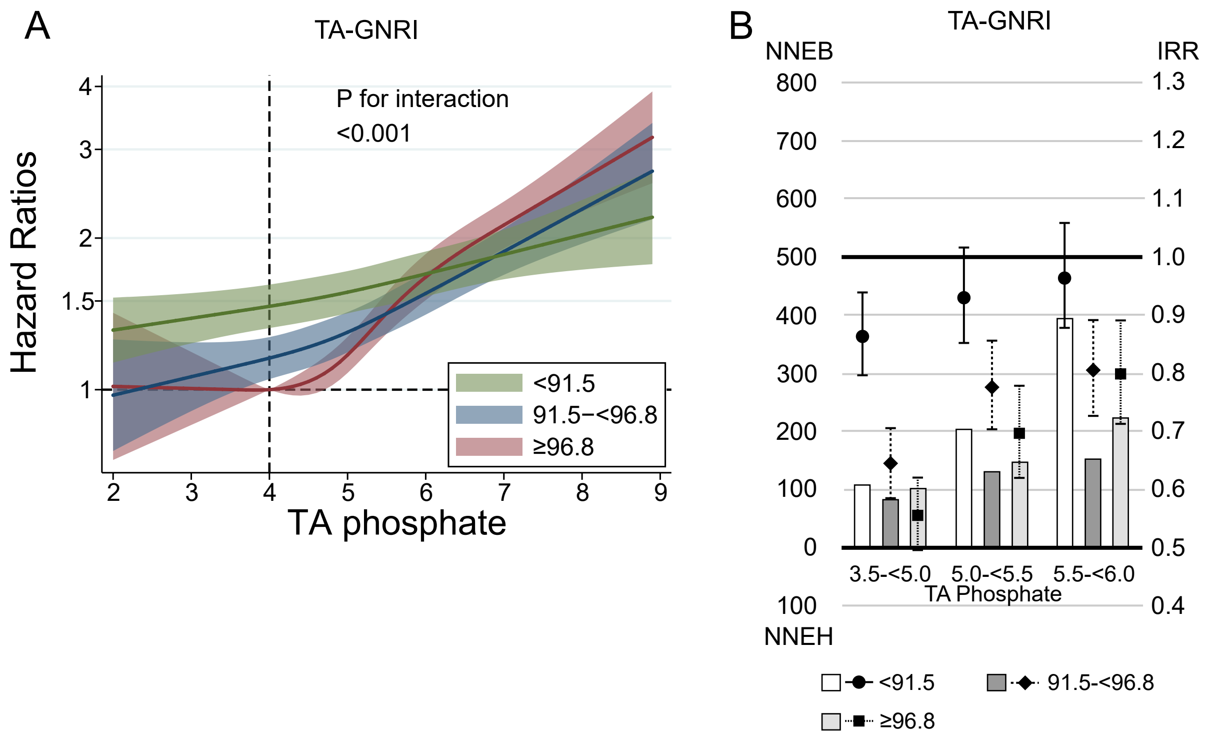


**Supplementary Figure 3: (A)** Relationship between time-averaged (TA) phosphate and (A) cardiovascular death according to TA geriatric nutritional risk index (GNRI) levels. (B) The one-year number needed to be exposed (NNE) and incidence rate ratio (IRR) for cardiovascular death in subgroups according to TA GNRI levels.

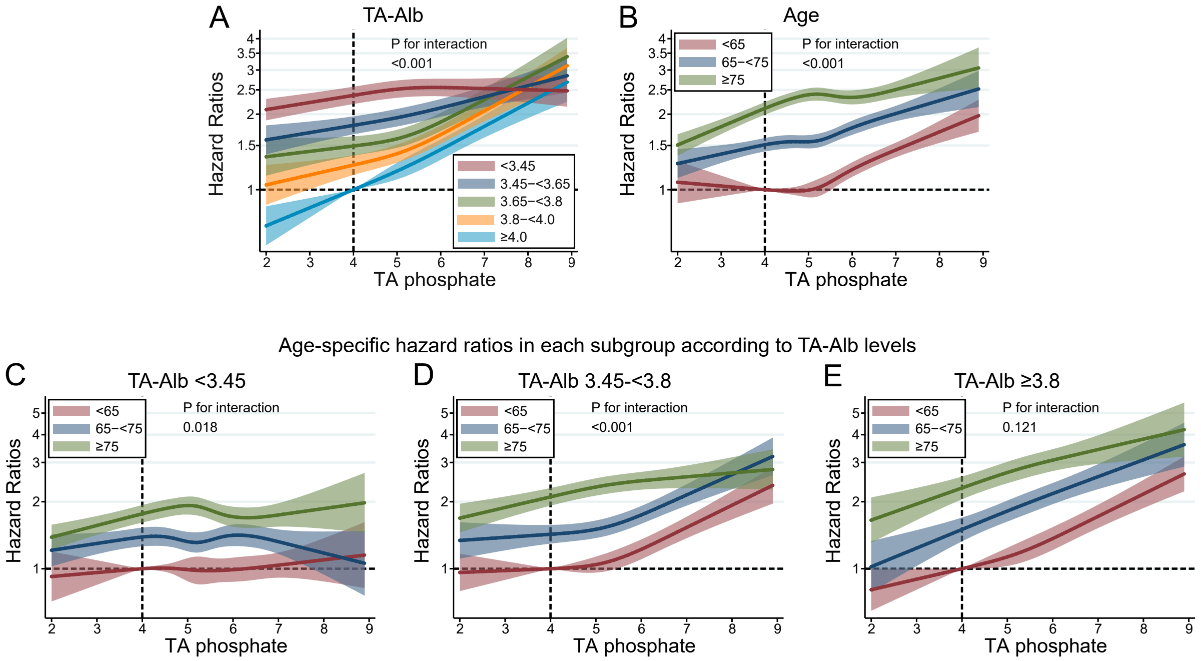


**Supplementary Figure 4.** Relationship between time-averaged (TA) phosphate and all-cause mortality in subgroups according to age or serum time-averaged albumin (TA-Alb) levels. (A) TA-Alb, (B) age, (C) age among patients with serum TA-Alb levels <3.45 g/dL, (D) age among patients with serum TA-Alb levels of 3.45–<3.8 g/dL, and (E) age among patients with serum TA-Alb levels ≥3.8 g/dL.

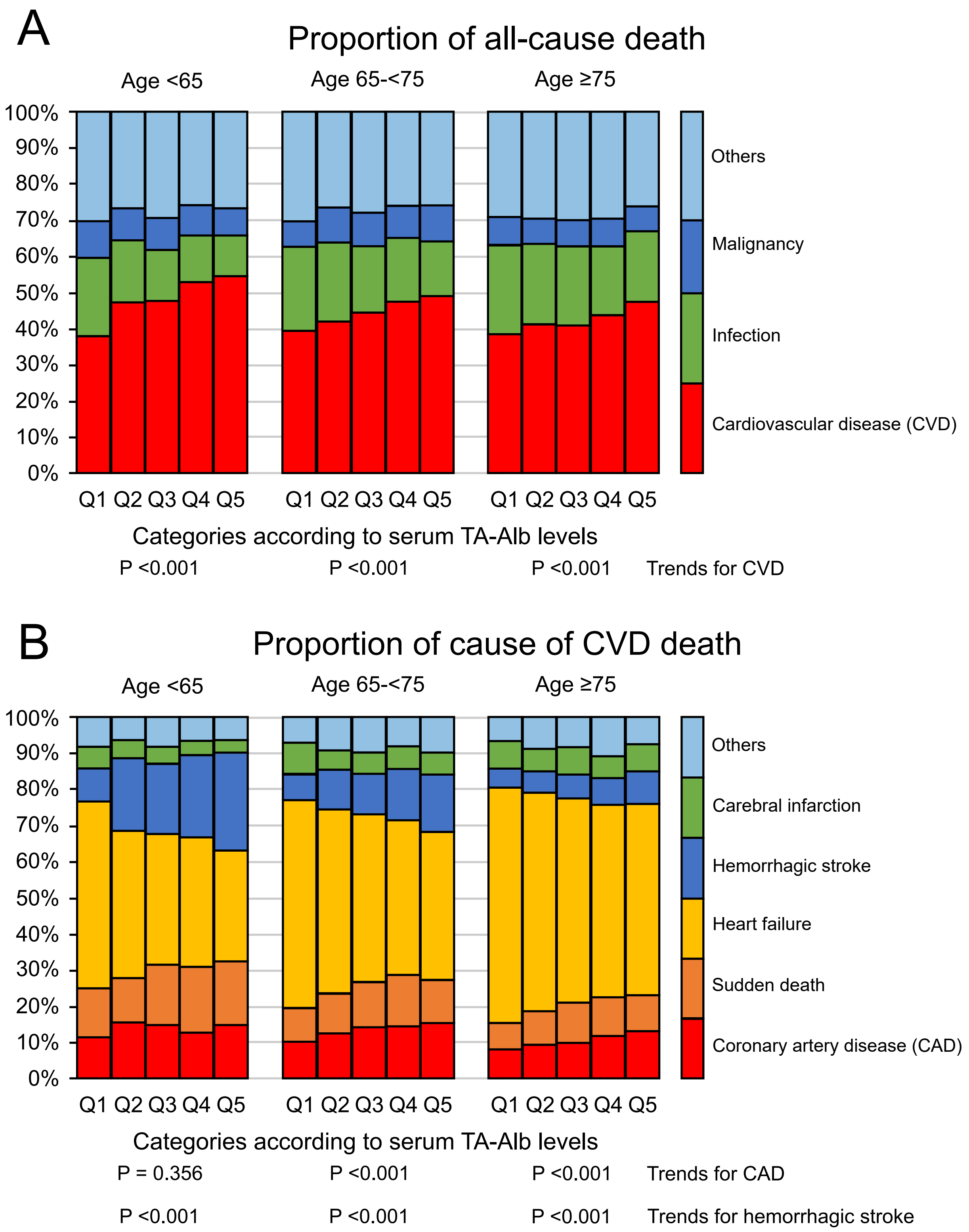


**Supplementary Figure 5.** Proportion of (A) all-cause death and (B) cardiovascular death in subgroups stratified age and serum time-averaged albumin (TA-Alb) levels. Range of quintiles of serum TA-Alb levels, as follows: Q1, <3.45 g/dL; Q2, 3.45–<3.65; Q3, 3.65–<3.8; Q4, 3.8–<4.0; and Q5, ≥4.0.

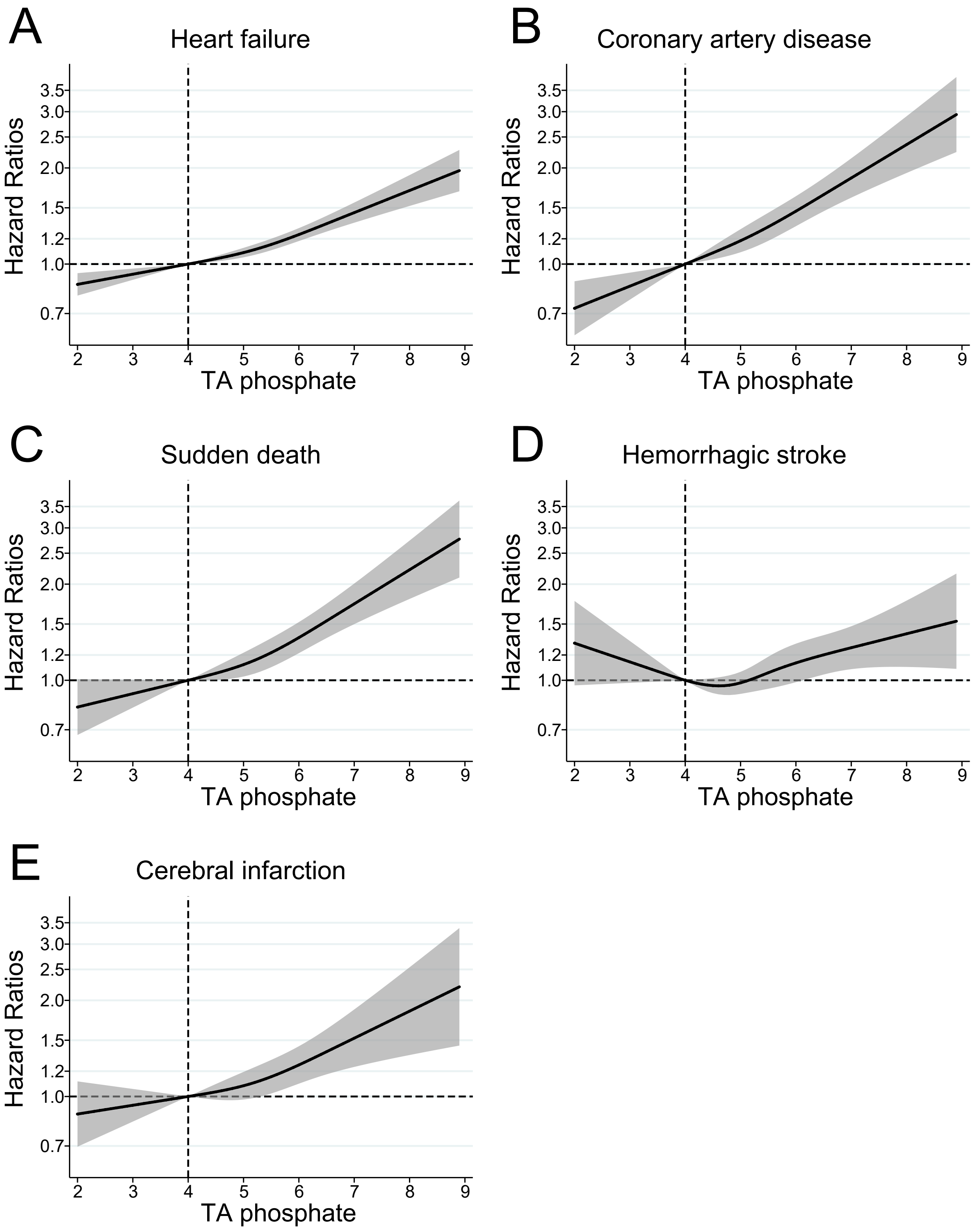


**Supplementary Figure 6.** Relationship between serum time-averaged (TA) phosphate and five main causes of cardiovascular death in all patients. (A) heart failure, (B) coronary artery disease, (C) sudden death, (D) hemorrhagic stroke, and (E) cerebral infarction.

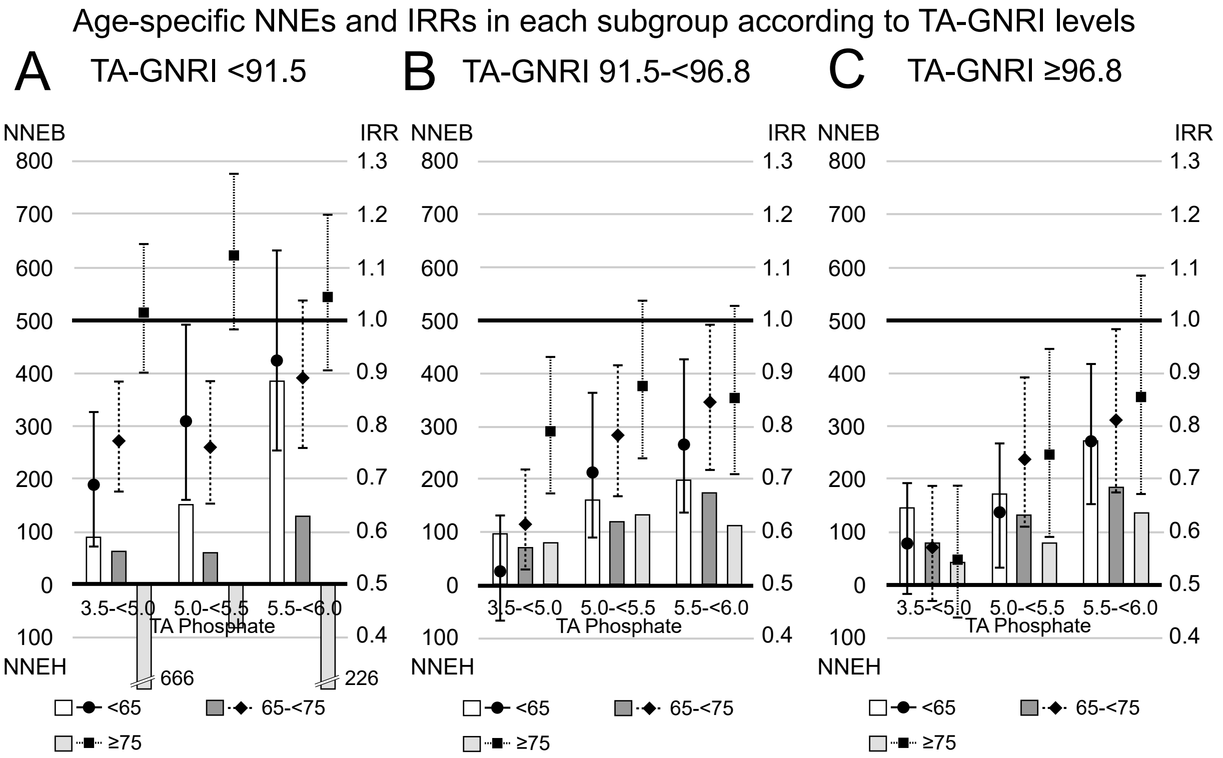


**Supplementary Figure 7:** The one-year number needed to be exposed (NNE) and incidence rate ratio (IRR) for cardiovascular death in subgroups according to age and TA geriatric nutritional risk index (GNRI) levels. Patients whose serum time-averaged (TA) phosphate levels maintained ≥6.0 mg/dL during the study were referenced in the analysis of IRR. The NNE to benefit (NNEB) or NNE to be harmed (NNEH) in individuals with baseline serum phosphate levels ≥6.0 mg/dL was calculated under the exposure of TA phosphate levels decreasing to each range (3.5–<5.0, 5.0–<5.5, or 5.5–<6.0 mg/dL). (A) age among patients with TA-GNRI levels <91.5, (B) age among patients with TA-GNRI levels of 91.5–<96.8, and (C) age among patients with TA-GNRI levels ≥96.8.

**Supplemental Table 1**. Hazard ratios and 95% confidence intervals for all-cause mortality based on serum time-averaged phosphate levels in subgroups according to the history of atherosclerotic cardiovascular disease (ACVD), diabetic nephropathy (DN), sex, and serum alkaline phosphatase (ALP) levels.

| | History of ACVD | | DN | | Sex | | ALP | |
|---|---|---|---|---|---|---|---|---|
| | ACVD | No ACVD | DN | No DN | Male | Female | Low ALP | High ALP |
| P for interaction | <0.001 | | <0.001 | | <0.001 | | 0.004 | |
| <3.5 | 0.99 (0.92, 1.07) | 0.98 (0.91, 1.05) | **1.12 (1.03, 1.21)*** | **0.88 (0.82, 0.94)*** | 0.97 (0.90, 1.03) | 0.98 (0.90, 1.07) | 1.00 (0.92, 1.09) | 0.97 (0.91, 1.04) |
| 3.5–<3.9 | ref | ref | ref | ref | ref | ref | ref | ref |
| 3.9–<4.3 | **1.16 (1.08, 1.24)*** | **1.09 (1.02, 1.17)*** | **1.15 (1.07, 1.24)*** | **1.11 (1.04, 1.18)*** | **1.14 (1.07, 1.22)*** | **1.08 (1.00, 1.17)*** | **1.12 (1.04, 1.21)*** | **1.13 (1.07, 1.21)*** |
| 4.3–<4.7 | **1.14 (1.07, 1.22)*** | **1.11 (1.04, 1.18)*** | **1.14 (1.06, 1.22)*** | **1.12 (1.05, 1.19)*** | **1.16 (1.09, 1.23)*** | 1.07 (1.00, 1.16) | **1.12 (1.04, 1.21)*** | **1.14 (1.07, 1.20)*** |
| 4.7–<5.1 | **1.15 (1.08, 1.23)*** | **1.12 (1.05, 1.20)*** | **1.15 (1.07, 1.24)*** | **1.13 (1.06, 1.20)*** | **1.15 (1.09, 1.23)*** | **1.12 (1.04, 1.21)*** | **1.11 (1.03, 1.19)*** | **1.18 (1.11, 1.25)*** |
| 5.1–<5.4 | **1.13 (1.05, 1.22)*** | **1.19 (1.11, 1.28)*** | **1.21 (1.12, 1.31)*** | **1.14 (1.07, 1.21)*** | **1.22 (1.15, 1.30)*** | **1.09 (1.01, 1.18)*** | **1.12 (1.04, 1.22)*** | **1.22 (1.14, 1.30)*** |
| 5.4–<5.8 | **1.19 (1.11, 1.27)*** | **1.18 (1.10, 1.26)*** | **1.26 (1.17, 1.36)*** | **1.14 (1.07, 1.21)*** | **1.25 (1.17, 1.33)*** | **1.10 (1.01, 1.19)*** | **1.18 (1.09, 1.28)*** | **1.20 (1.13, 1.28)*** |
| 5.8–<6.3 | **1.22 (1.13, 1.32)*** | **1.21 (1.13, 1.30)*** | **1.29 (1.19, 1.39)*** | **1.17 (1.10, 1.26)*** | **1.27 (1.19, 1.35)*** | **1.16 (1.07, 1.27)*** | **1.24 (1.14, 1.34)*** | **1.23 (1.15, 1.32)*** |
| 6.3–<7.0 | **1.31 (1.20, 1.42)*** | **1.38 (1.28, 1.49)*** | **1.42 (1.30, 1.55)*** | **1.30 (1.20, 1.39)*** | **1.41 (1.32, 1.51)*** | **1.28 (1.17, 1.41)*** | **1.42 (1.31, 1.55)*** | **1.31 (1.22, 1.41)*** |
| 7.0– | **1.53 (1.39, 1.68)*** | **1.62 (1.48, 1.76)*** | **1.71 (1.55, 1.88)*** | **1.48 (1.37, 1.61)*** | **1.67 (1.55, 1.81)*** | **1.44 (1.30, 1.60)*** | **1.61 (1.46, 1.77)*** | **1.61 (1.48, 1.75)*** |

* P <0.05

**Supplemental Table 2**. Hazard ratios and 95% confidence intervals for all-cause mortality based on serum time-averaged phosphate levels in subgroups according to age and serum time-averaged albumin levels.

| | Time-averaged albumin | | | | |
|---|---|---|---|---|---|
| | <3.45 | 3.45-<3.65 | 3.65-<3.8 | 3.8-<4.0 | ≥4.0 |
| P for interaction | | | <0.001 | | |
| <3.5 | 0.99 (0.93, 1.06) | 1.01 (0.89, 1.15) | 1.01 (0.83, 1.22) | 0.88 (0.71, 1.09) | 0.94 (0.68, 1.28) |
| 3.5–<3.9 | ref | ref | ref | ref | ref |
| 3.9–<4.3 | **1.15 (1.07, 1.22)*** | 1.10 (0.99, 1.22) | 1.02 (0.88, 1.18) | 1.11 (0.95, 1.31) | 1.03 (0.81, 1.31) |
| 4.3–<4.7 | **1.11 (1.04, 1.19)*** | **1.15 (1.04, 1.26)*** | 1.08 (0.94, 1.24) | 1.10 (0.94, 1.29) | 1.15 (0.91, 1.45) |
| 4.7–<5.1 | **1.13 (1.05, 1.20)*** | **1.15 (1.04, 1.27)*** | 1.08 (0.94, 1.24) | 1.16 (1.00, 1.35) | 1.15 (0.91, 1.44) |
| 5.1–<5.4 | **1.11 (1.03, 1.20)*** | **1.17 (1.06, 1.30)*** | 1.14 (0.99, 1.31) | **1.26 (1.08, 1.47)*** | **1.36 (1.07, 1.71)*** |
| 5.4–<5.8 | **1.08 (1.01, 1.17)*** | **1.23 (1.11, 1.36)*** | **1.19 (1.04, 1.37)*** | **1.27 (1.09, 1.48)*** | **1.37 (1.09, 1.72)*** |
| 5.8–<6.3 | 1.07 (0.99, 1.16) | **1.19 (1.08, 1.33)*** | **1.32 (1.15, 1.53)*** | **1.34 (1.14, 1.56)*** | **1.63 (1.29, 2.05)*** |
| 6.3–<7.0 | **1.10 (1.00, 1.20)*** | **1.39 (1.25, 1.56)*** | **1.54 (1.32, 1.80)*** | **1.47 (1.25, 1.74)*** | **1.77 (1.39, 2.24)*** |
| 7.0– | 1.08 (0.97, 1.20) | **1.58 (1.38, 1.81)*** | **1.89 (1.59, 2.24)*** | **2.09 (1.75, 2.48)*** | **2.13 (1.67, 2.73)*** |

| | Age | | |
|---|---|---|---|
| | <65 | 65-<75 | ≥75 |
| P for interaction | | <0.001 | |
| <3.5 | 0.88 (0.75, 1.03) | 1.01 (0.91, 1.12) | 0.98 (0.92, 1.05) |
| 3.5–<3.9 | ref | ref | ref |
| 3.9–<4.3 | 1.01 (0.87, 1.17) | **1.12 (1.02, 1.23)*** | **1.12 (1.06, 1.19)*** |
| 4.3–<4.7 | 0.96 (0.84, 1.11) | **1.10 (1.01, 1.21)*** | **1.14 (1.07, 1.21)*** |
| 4.7–<5.1 | 1.12 (0.89, 1.17) | 1.08 (0.99, 1.18) | **1.16 (1.09, 1.23)*** |
| 5.1–<5.4 | 1.00 (0.86, 1.15) | **1.14 (1.04, 1.26)*** | **1.20 (1.12, 1.28)*** |
| 5.4–<5.8 | **1.17 (1.02, 1.34)*** | **1.19 (1.08, 1.30)*** | **1.13 (1.06, 1.21)*** |
| 5.8–<6.3 | **1.21 (1.06, 1.39)*** | **1.26 (1.15, 1.39)*** | **1.13 (1.06, 1.22)*** |
| 6.3–<7.0 | **1.42 (1.23, 1.63)*** | **1.38 (1.25, 1.53)*** | **1.20 (1.11, 1.31)*** |
| 7.0– | **1.78 (1.54, 2.05)*** | **1.55 (1.38, 1.74)*** | **1.25 (1.12, 1.39)*** |

* P <0.05

**Supplemental Table 3.** Age-specific hazard ratios and 95% confidence intervals for all-cause mortality based on serum time-averaged phosphate levels in subgroups according to serum time-averaged albumin levels.

| | Time-averaged albumin <3.45 | | | Time-averaged albumin 3.45-<3.8 | | |
|---|---|---|---|---|---|---|
| | <65 | 65-<75 | ≥75 | <65 | 65-<75 | ≥75 |
| P for interaction | | 0.012 | | | <0.001 | |
| <3.5 | 0.83 (0.67, 1.03) | 0.94 (0.82, 1.07) | 1.01 (0.93, 1.10) | 0.98 (0.71, 1.36) | 1.17 (0.95, 1.43) | 0.96 (0.84, 1.10) |
| 3.5–<3.9 | ref | ref | ref | ref | ref | ref |
| 3.9–<4.3 | 0.96 (0.77, 1.20) | 1.08 (0.95, 1.24) | **1.17 (1.08, 1.27)*** | 0.92 (0.71, 1.19) | **1.20 (1.02, 1.42)*** | 1.04 (0.93, 1.15) |
| 4.3–<4.7 | 0.94 (0.75, 1.16) | 0.99 (0.87, 1.13) | **1.16 (1.07, 1.25)*** | 0.92 (0.72, 1.17) | **1.27 (1.09, 1.47)*** | 1.10 (0.99, 1.22) |
| 4.7–<5.1 | 1.02 (0.83, 1.27) | 0.98 (0.86, 1.12) | **1.16 (1.07, 1.26)*** | 0.98 (0.78, 1.23) | **1.20 (1.03, 1.39)*** | **1.13 (1.02, 1.25)*** |
| 5.1–<5.4 | 0.96 (0.77, 1.21) | 0.99 (0.86, 1.14) | **1.15 (1.05, 1.26)*** | 0.92 (0.72, 1.17) | **1.30 (1.11, 1.52)*** | **1.18 (1.06, 1.31)*** |
| 5.4–<5.8 | 1.02 (0.82, 1.28) | 1.03 (0.89, 1.19) | 1.07 (0.98, 1.18) | 1.24 (0.99, 1.56) | **1.39 (1.19, 1.62)*** | **1.13 (1.01, 1.26)*** |
| 5.8–<6.3 | 1.00 (0.80, 1.26) | 1.03 (0.88, 1.30) | 1.05 (0.95, 1.17) | 1.16 (0.92, 1.46) | **1.44 (1.23, 1.69)*** | **1.17 (1.04, 1.32)*** |
| 6.3–<7.0 | 1.08 (0.85, 1.38) | 1.14 (0.97, 1.34) | 1.01 (0.89, 1.15) | **1.39 (1.10, 1.75)*** | **1.65 (1.39, 1.95)*** | **1.34 (1.17, 1.53)*** |
| 7.0– | **1.25 (1.05, 1.74)*** | 0.86 (0.69, 1.32) | 1.07 (0.90, 1.26) | **1.65 (1.30, 2.10)*** | **2.09 (1.73, 2.52)*** | **1.22 (1.02, 1.46)*** |

| | Time-averaged albumin ≥3.8 | | |
|---|---|---|---|
| | <65 | 65-<75 | ≥75 |
| P for interaction | | 0.232 | |
| <3.5 | 1.24 (0.82, 1.89) | 0.89 (0.64, 1.23) | 0.87 (0.68, 1.12) |
| 3.5–<3.9 | ref | ref | ref |
| 3.9–<4.3 | 1.33 (0.97, 1.83) | 1.09 (0.86, 1.38) | 1.00 (0.83, 1.21) |
| 4.3–<4.7 | 1.20 (0.88, 1.64) | 1.19 (0.95, 1.49) | 1.05 (0.87, 1.26) |
| 4.7–<5.1 | 1.29 (0.95, 1.74) | **1.26 (1.01, 1.56)*** | 1.08 (0.90, 1.29) |
| 5.1–<5.4 | 1.36 (1.00, 1.94) | **1.36 (1.09, 1.71)*** | **1.26 (1.04, 1.52)*** |
| 5.4–<5.8 | **1.49 (1.10, 2.00)*** | **1.37 (1.09, 1.71)*** | 1.19 (0.99, 1.44) |
| 5.8–<6.3 | **1.73 (1.29, 2.33)*** | **1.57 (1.25, 1.96)*** | 1.20 (0.98, 1.46) |
| 6.3–<7.0 | **1.99 (1.47, 2.68)*** | **1.63 (1.28, 2.06)*** | **1.26 (1.02, 1.57)*** |
| 7.0– | **2.53 (1.87, 3.43)*** | **2.10 (1.63, 2.71)*** | **1.62 (1.26, 2.07)*** |

* P <0.05
